# Quercetin Suppresses Twist to Induce Apoptosis in MCF-7 Breast Cancer Cells

**DOI:** 10.1371/journal.pone.0141370

**Published:** 2015-10-22

**Authors:** Santhalakshmi Ranganathan, Devaraj Halagowder, Niranjali Devaraj Sivasithambaram

**Affiliations:** 1 Department of Biochemistry, University of Madras, Guindy Campus, Chennai, India; 2 University Grants commission, New Delhi, India; University of Nebraska Medical Center, UNITED STATES

## Abstract

Quercetin is a dietary flavonoid which exerts anti-oxidant, anti-inflammatory and anti-cancer properties. In this study, we investigated the anti-proliferative effect of quercetin in two breast cancer cell lines (MCF-7 and MDA-MB-231), which differed in hormone receptor. IC_50_ value (37μM) of quercetin showed significant cytotoxicity in MCF-7 cells, which was not observed in MDA-MB-231 cells even at 100μM of quercetin treatment. To study the response of cancer cells to quercetin, with respect to different hormone receptors, both the cell lines were treated with a fixed concentration (40μM) of quercetin. MCF-7 cells on quercetin treatment showed more apoptotic cells with G1 phase arrest. In addition, quercetin effectively suppressed the expression of CyclinD1, p21, Twist and phospho p38MAPK, which was not observed in MDA-MB-231 cells. To analyse the molecular mechanism of quercetin in exerting an apoptotic effect in MCF-7 cells, Twist was over-expressed and the molecular changes were observed after quercetin administration. Quercetin effectively regulated the expression of Twist, in turn p16 and p21 which induced apoptosis in MCF-7 cells. In conclusion, quercetin induces apoptosis in breast cancer cells through suppression of Twist via p38MAPK pathway.

## Introduction

Breast cancer is the most frequently diagnosed cancer among women in India and worldwide [1]. The breast cancer burden in India has almost reached about 2/3^rd^ of United States and it is steadily increasing. It is estimated that there are nearly 1.5–2 million cancer cases in India. The mortality rate in India is huge (1 in 2 newly diagnosed cases) [2], irrespective of the treatment they get.

Progression of breast cancer is a multistep process, which involves hormones and genes like tumor suppressor genes, oncogenes and recently it has been shown that developmental genes are also involved [3]. Estrogen is a hormone which fuels the cancer cells to grow. Similarly, the genes involved in the development of embryo are later found to be involved in progression of cancer. One such gene, widely spoken in the last decade is “twist”, which is essential in embryonic developmental stage. The same is involved in cancer metastasis by down-regulating E-Cadherin, thereby inducing cell movement and invasion. Twist protein is a bHLH transcription factor which binds to E-box responsive element (CANNTG) and behaves either as a transcription repressor or activator, depending on the cellular context [4–6]. It is known that twist is expressed in various types of cancer [7]. Twist is over-expressed in several kinds of tumors like breast, uterus, lung, liver, hepatocellular, prostrate, gastric carcinoma and melanomas [8–12].

There is an emerging need for natural drugs, because cancer cells show resistance and decreased sensitivity to the available chemotherapeutic agents. Although the current chemotherapeutics are able to inhibit or kill tumors, the issues of toxicity and side effects stay behind in restricting the clinical application of these drugs. Any natural compound, which could kill the cancer cells and has no or least effect on normal cells is considered for cancer therapeutic strategies. One such natural flavonoid is quercetin which is known to have antioxidant and anti-inflammatory properties.

Quercetin is a natural flavonoid, present in barks of many plants, fruits and vegetables [13]. It is a phytoestrogen, which structurally mimics endogenous estrogen 17beta-estradiol [14, 15]. Several *in vivo* and *in vitro* studies showed the inhibitory effect of quercetin in various cancer cells including breast [16, 17]. Previous studies revealed that quercetin regulated the expression of an oncogene c-myc [18]. The present study aimed to seek the capability of quercetin in regulating twist in two different cell lines which differ in their hormone receptor (MCF-7 and MDA-MB-231). MCF-7 cell line is a widely studied model for hormone-dependant human breast cancer. These cells contain functional estrogen receptors and show a pleotropic response to estrogen [19, 20, 21]. Estrogen stimulates proliferation of these cells in vitro [22, 23]. In contrast, MDA-MB-231 cell line does not express estrogen receptor and it exhibits an estrogen-independent breast cancer model [24]. Recent studies revealed that quercetin is able to prevent and treat cancer by inhibiting the growth of cancer cells [25, 26]. To determine the ability of quercetin to treat breast cancer, we investigated the effect of quercetin on two different human breast cancer cell lines. Furthermore, we assessed the growth inhibitory effect of quercetin in MCF-7 cells by observing changes in twist protein expression.

## Materials and Methods

### Cell culture and drug treatment

MCF- 7 and MDA MB 231 cells obtained from National Centre for Cell Science (NCCS), Pune, India, were grown in DMEM with 2 mM glutamine, 10% Fetal Bovine Serum (FBS), 1% antibiotics (Gibco-Bethesda Research Laboratories, Gaithersburg, MD) at 37°C in 5% CO_2_ atmosphere.

Quercetin was purchased from Sigma Aldrich Chemicals Pvt Ltd and dissolved in 100% DMSO. The final concentration of DMSO in the culture medium never exceeded 0.2% (v/v), and the same concentration was present in control experiments.

### Cell proliferation assay

Cell proliferation/ viability were determined using MTT assay. Cells were seeded in 96 well plate at 5x10^2^ cells/well. A day after seeding, the culture medium was changed and quercetin was added at different doses. The cells exposed to quercetin were treated with methyl thiazolyl tetrazolium (MTT). Four hours later, DMSO was added to each well to dissolve formazan crystals and absorbance was recorded at 570nm in a microplate reader.

### Growth rate and flow cytometry analysis

Cells were seeded in 60mm dishes at 2x10^5^ cells/dish. A day after inoculation, the culture medium was changed and quercetin was added at different doses. At various experimental intervals, the number of viable cells was determined by the Trypan blue dye exclusion test (S1 Fig and S2 Fig).

The effect of quercetin on cell cycle was studied on actively growing cells: 2x10^5^ cells were seeded in 6 cm dishes, and after 24 h, the drug was added at different concentrations. The cells treated with quercetin were fixed at different time points using 70% ethanol and stored overnight at 4°C. The fixed cells were washed with 1X PBS and stained with Propidium Iodide (PI) for analysis. Cell cycle analysis was performed using flow cytometer (BD FACSCalibur).

### Dual staining

The cells were seeded on either cover slip or in chambered slide (1000 cells per well) and treated with quercetin on the next day. After the treatment, the cells were washed twice with 1X PBS and stained with 100μl of Ethidium Bromide (EtBr) and Acridine Orange (AO) solution (1:1) and mixed gently. Each sample was mixed just prior to microscopic examination and quantified immediately. The slides were placed on the platform and cell staining was observed in a fluorescence microscope (Olympus-BH2, Lake success, NY, USA). The percentage of apoptotic cells = (total number of apoptotic cells/total number of cells counted) x 100.

### Protein extraction and immunoblotting analysis

After treatment, cells (2x10^5^ / 6cm dish) were washed with phosphate buffered saline and disrupted by addition of 60–80 *μ*l of lysis buffer [25mM Hepes, 150mM NaCl, 1%NP40,10mM MgCl_2_,10% Glycerol, 1mM EDTA, Aprotinin 0.5*μ*g/ml,1mMNa_3_VO_4_,1mM NaF, 1mM PMSF]. The disrupted cells were spun at maximum speed for 15 min; the resulting supernatant protein was collected. Equal amounts of protein (50*μ*g) were subjected to 10% SDS-PAGE and samples were transferred electrophoretically to polyvinylidenedifluoride membranes (Millipore, Bedford, MA) in a transfer buffer containing 25mM Tris, 192 mM glycine and 20% (v/v) methanol. Membranes were blocked overnight with 5% fat free dry milk in phosphate buffered saline containing 0.05% Tween-20. Immunodetection was performed using antibodies to Twist (Santa Cruz Biotechnology), Cyclin D1, p38 MAPK and β actin (Cell signalling technology). Membranes were incubated over night with each antibody at 4°C and later incubated for 1.5 h with HRP-conjugated secondary antibody. Proteins were visualised using enhanced chemiluminescent reagent kit (Amersham, ECL advance, Western blotting detection kit, UK), as per the manufacturers protocol. Quantification of the Western blots was performed using ImageJ software.

### Immunofluorescence

MCF-7 and MDA-MB-231 cells (2 x10^4^) were plated onto poly-D-lysine-treated four-well chamber glass slides (Falcon). Following growth, the cells were fixed and blocked with 1% BSA. After blocking, the cells were washed and primary antibody for Twist (1:100, FITC-conjugated anti-rabbit) was added and incubated for 1 hour at room temperature. Cells were then washed and mounted with aqueous mounting medium, dried and examined using fluorescence microscope.

### Reverse transcription (RT)–PCR

Cells were grown for 24 h, and then treated for 24 h with quercetin. At the end of the treatment, RNA was extracted using Trizol Reagent (Qiagen) according to the manufacturer’s protocol. The RNA concentration was quantified by Biophotometer (Eppendrof, Germany). cDNAs were synthesized from RNA using M-MLV reverse transcriptase (GeNeiTM, Bangalore). Specific primer sequences (S1 Table) were used to amplify gene transcripts. The reaction was amplified in a thermal cycler (Eppendrof, Germany). The PCR conditions were for 5min of initial denaturation at 94°C and 35 cycles consisting of 94°C for 30 sec; annealing at respective Tm (mentioned in S1 Table) for 30 sec; elongation at 72°C for 1min and final extension at 72°C for 10 min. The amplified products (10μl) were electrophoresed on 1% agarose gel with ethidium bromide and visualised under UV light (Vilber Lourmat, France)

### Transfection of twist plasmid

The cells were seeded in 6 well plate with the desired density (2x10^5^ cells) and allowed to grow for 24h or till it became 60% confluent. To transfect the cells with plasmid DNA, 1μg of purified DNA was diluted with 100μl of serum-free DMEM. To the diluted DNA, 2μl of the transfection (TurboFect) reagent was added and mixed well. The DNA-TurboFect mixture was incubated for 20 min at room temperature. 100μl of the reagent mixture was added drop-wise to each well without removing the growth medium from the cells. The reagent mixture was mixed well by gently rocking the plate and incubated for 24–48h at 37°C in a CO_2_ incubator. After 48h, the cells were grown in selective medium for 20days. The transgene expression was seen after 48h of transfection. However the protein expression was seen even after 10 days of transfection.

### Soft agar colony formation assay

Normal cells will not grow in soft agar due to anoikis, while transformed cells will grow and form colonies. In sterile 50ml tubes, DMEM, FBS, sterile water were pre-warmed at 65°C. The noble agar (1.8%) was melted and left at 55°C to 65°C. The agar was quickly added to the medium tube and vortexed at a low speed prior to pouring into the dish. The dishes were left inside the hood for at least 45 min for the agar to solidify or kept at 37°C overnight. Overlay: The media, serum and water were pre-warmed at 42°C. The agar was boiled and left at 65°C. Later, the agar was quickly added to the medium, mixed gently by vortex without forming bubbles. The cells were counted and around 5000 cells were added to the medium and gently poured on the underlay. The overlay was left inside the hood till it solidified and incubated at 37°C at 5% CO_2_. The colonies could be seen within 15 to 20 days of incubation.

### Transmission electron microscopy

The cell suspensions were centrifuged and the pellets were fixed with 2.5% glutaraldehyde solution (pH 7.3) for about 2 hours at 4°C. The cell suspensions were then rinsed twice with 0.1M sodium cacodylate buffer and post-fixed with 1% osmium tetraoxide solution (pH 7.3) and incubated for 35min at 4°C. The cells were then dehydrated in a graded series of alcohol for 5 minutes each. The dehydrated pellets were embedded three times with propylene oxide for 1 hour each and infiltrated with a resin/propylene oxide mixture at a 1:1 ratio for 2 hours and then with resin only for 12 hours at ambient temperature. The cells were pelleted in pure epoxy resin and baked at 60°C overnight. The cells were embedded in pure embedding medium in beam capsule. The blocks were trimmed and the ultrathin sections were stained with uranyl acetate and counterstained with lead citrate. They were examined with the JEOL 1200 II transmission electron microscope.

### Statistical analysis

Statistical data analysis was done using statistical package for the social sciences (SPSS) version 16. The values are expressed as mean±SD. Hypothesis testing method included one-way analysis of variance (ANOVA) followed by post hoc testing performed with least significant difference test. For all comparisons, differences were considered statistically significant at p<0.05.

## Results

### Investigation of quercetin cytotoxicity in breast cancer cells

**Fig 1:**
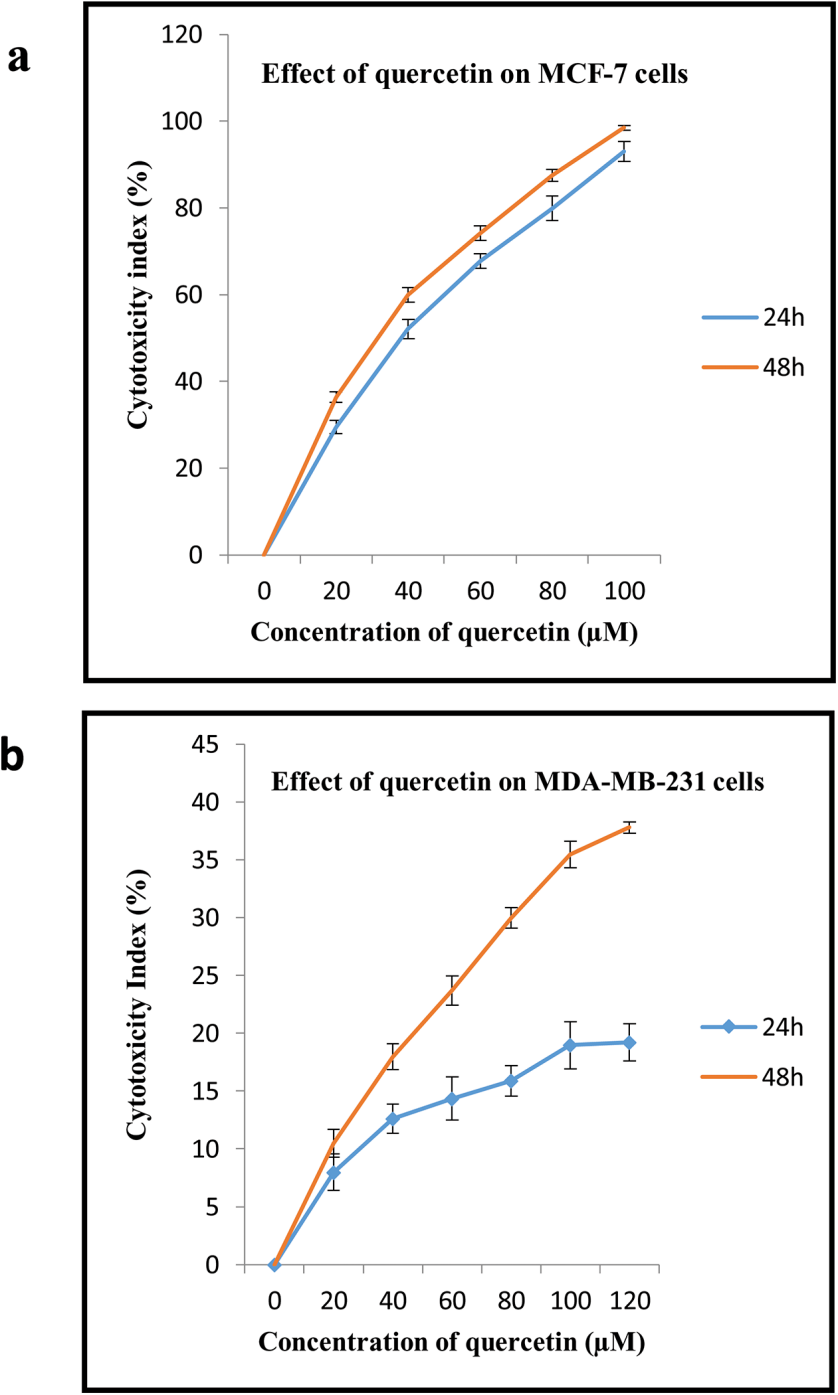
Effect of quercetin on breast cancer cell lines. A. Cytotoxicity effect of various concentrations of quercetin on MCF-7 cells. Dose dependent cytotoxicity effect of quercetin was represented in the graph. The IC50 value was 37μM of quercetin at 24h exposure. B. Cytotoxicity effect of various concentrations of quercetin on MDA-MB-231 cells. Dose dependent cytotoxicity effect of quercetin was represented in the graph. Cells did not show significant cytotoxicity at lower dose of quercetin at 24h of exposure. The significant values represent mean ± SD of three separate experiments, each consisting of duplicate cultures.

The cytotoxicity of quercetin in breast cancer cell lines MCF-7 and MDA-MB-231was evaluated by MTT assay. MCF-7 cells exhibits estrogen receptor (ER) and MDA-MB-231cells exhibits epidermal growth factor (EGFR) receptor. Cells were treated with various doses of quercetin (10μM to 100μM) for 24h and 48h. MCF-7 cells were sensitive to quercetin with an IC_50_ value of 37μM, indicating cytotoxicity effect of quercetin in ER presenting cells (Fig 1A). However, MDA-MB-231 cells did not show significant cytotoxicity to quercetin, though at 48h of 100μM quercetin treatment less than 40% of cells showed cytotoxicity (Fig 1B). We restricted our study, to lower concentrations of quercetin, as few other studies state that higher concentrations of quercetin become carcinogenic and induce risk of leukemia in young children [27, 28]. Hence, we fixed up a minimal dosage of 40μM of quercetin for both the cell lines.

### Quercetin induced apoptosis in MCF-7 cells

**Fig 2:**
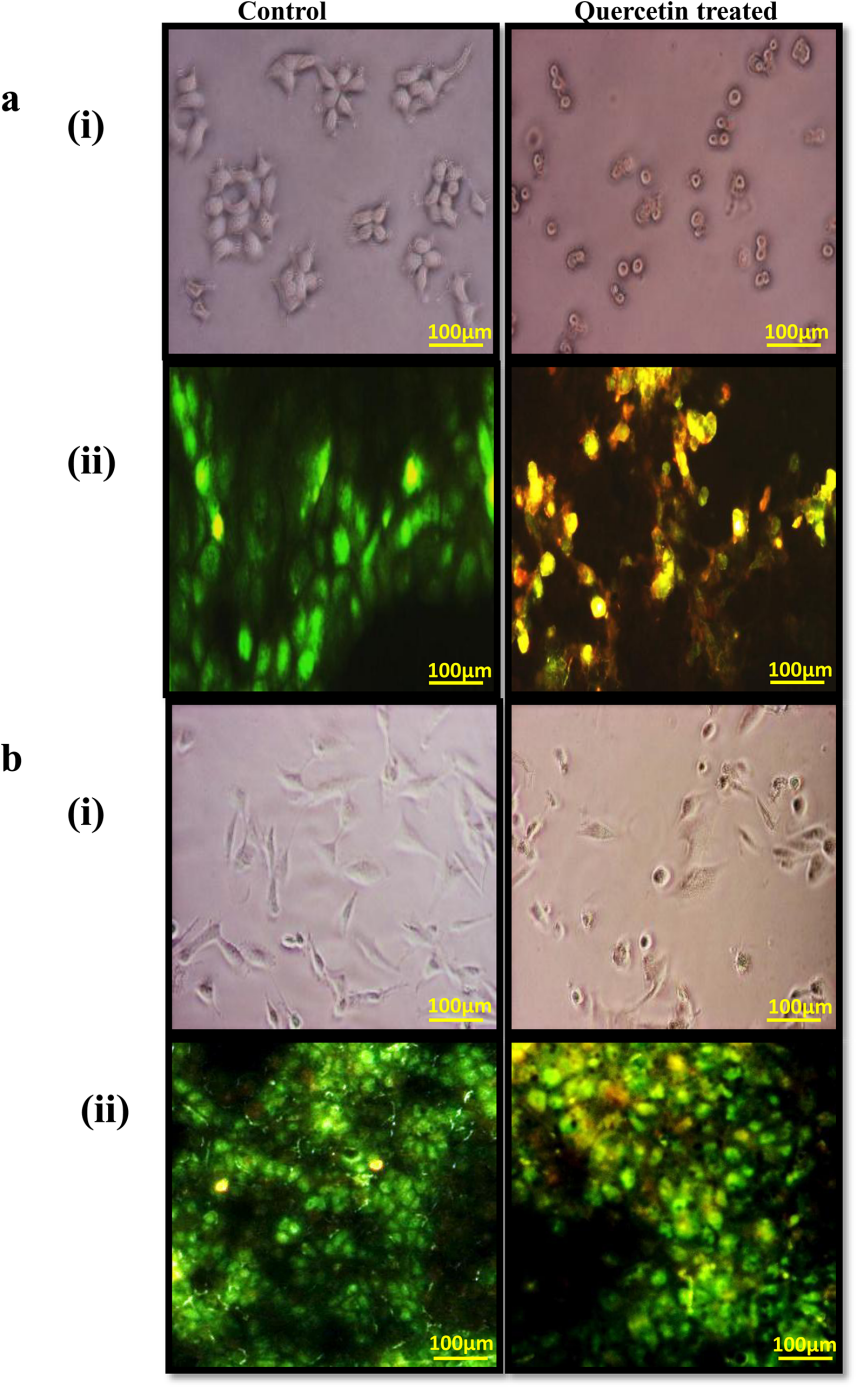
Cell pictures show the cell morphology and viability of MCF-7 and MDA-MB-231 control and quercetin treated cells. a(i) MCF-7 cells cultured with and without quercetin for 24h were examined for changes in cell morphology and photographed using a phase-contrast microscope. a(ii) MCF-7 cells with and without quercetin for 24h were examined for the viability of cells by dual staining. b(i) MDA-MB-231 cells cultured with and without quercetin for 24h were examined for changes in cell morphology and photographed using a phase-contrast microscope. b(ii) MDA-MB-231 cells with and without quercetin for 24h were examined for the viability of cells by dual staining. The green stained are viable cells and the yellowish orange stained are damaged/apoptotic cells.

Cytotoxicity of selected dosage of quercetin was analysed by dual staining assay with acridine orange and ethidium bromide. The quercetin treated MCF-7 cells (morphology) were not as viable as control (Fig 2A(i)), appearance of yellowish orange cells indicates DNA damage, which is hall mark of dual staining analysis (Fig 2A(ii)). This indicates that the cytotoxicity of quercetin in MCF-7 cells was due to apoptosis. However, MDA-MB-231 cells (morphology) were as viable as control (Fig 2B(i)) in the selected dosage of quercetin. The dual staining also depicts the viability of MDA-MB-231 cells after quercetin treatment (Fig 2B (ii)).

**Fig 3:**
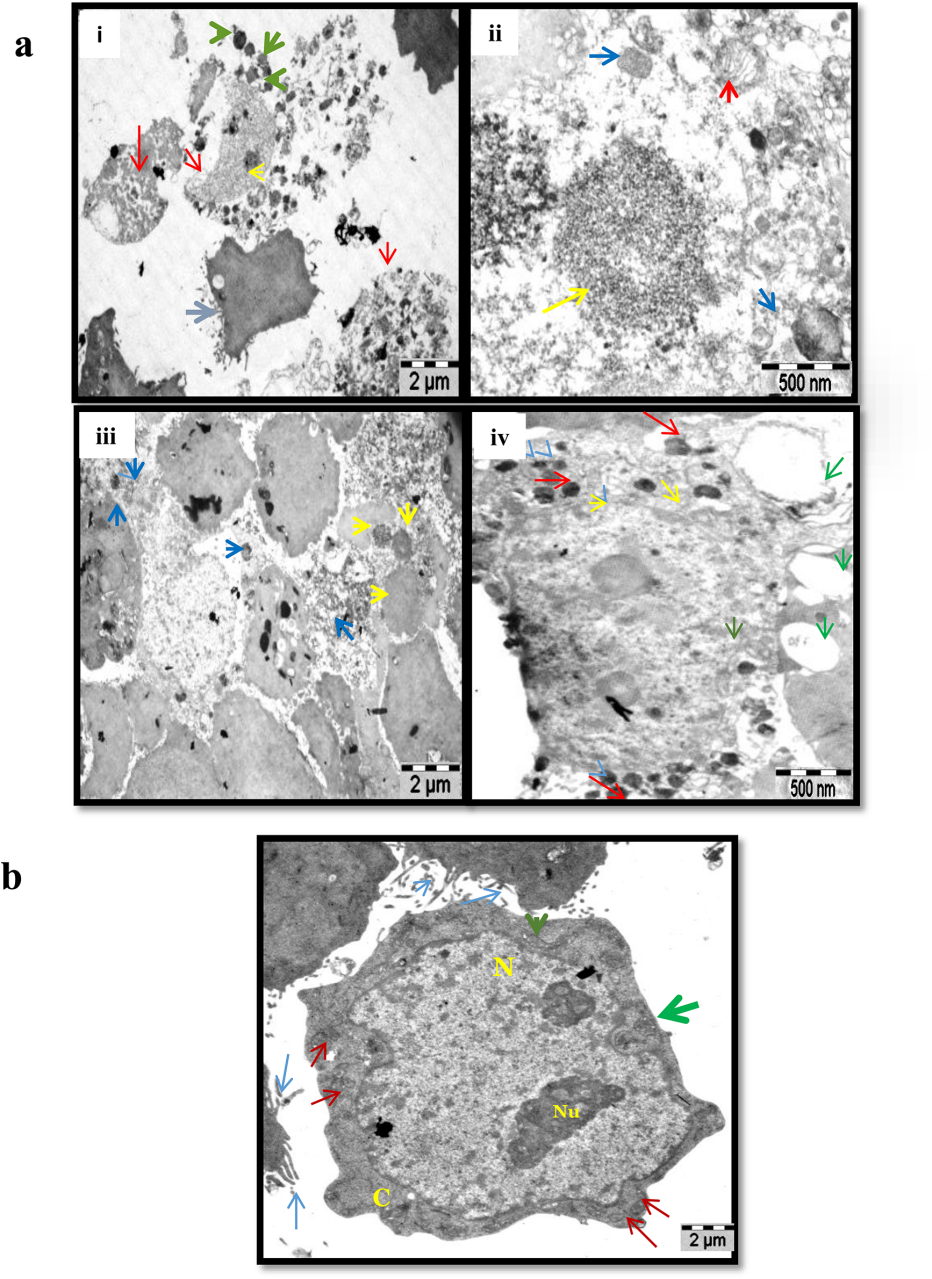
TEM picture represents the morphology and organelles of MCF-7 control and quercetin treated cells. A. TEM analysis showing the structural changes and damages occur on treatment with quercetin. Picture (i) shows a undamaged cell (blue arrow) in the middle along with the cells undergoing apoptotic necrosis (red arrow), chromatin condensation inside the nucleus (yellow arrow) and autophagosomes (green arrow). Magnification at 500nm (ii) shows the condensation of the chromatin (yellow arrow), autophagosomes (blue arrow) and ER vesiculation (red arrow). (iii) Represents cells at lower magnification showing plenty of chromatin condensation (yellow) and autophagosis (blue) in group. (iv) Represents a single damaged cell showing huge number of autophagosis (red arrow), nuclear membrane showing starting of hetero chromatin (yellow), large number of vesicles in the cytosol (green arrow). B. The picture shows a single cell with microvilli (blue arrow), nucleus (N) present at the center of the cell, nucleoli (Nu), mitochondria (red arrow) (shows active energy production), structured Golgi apparatus (green arrow) and endoplasmic reticulum (orange arrow) finely organised. All these events represent a cell in active state.

The quercetin induced apoptosis in MCF-7 cells was confirmed by Transmission Electron Microscopy (TEM), where the cells showed more number of damaged cells with chromatin condensation and autophagosis (Fig 3B), in contrast to control MCF-7 cells which showed a normal architecture with structured nucleus, nucleolus and microvilli representing a proliferating cell (Fig 3A).

### Quercetin induced cell cycle arrest in MCF-7 cells

**Fig 4:**
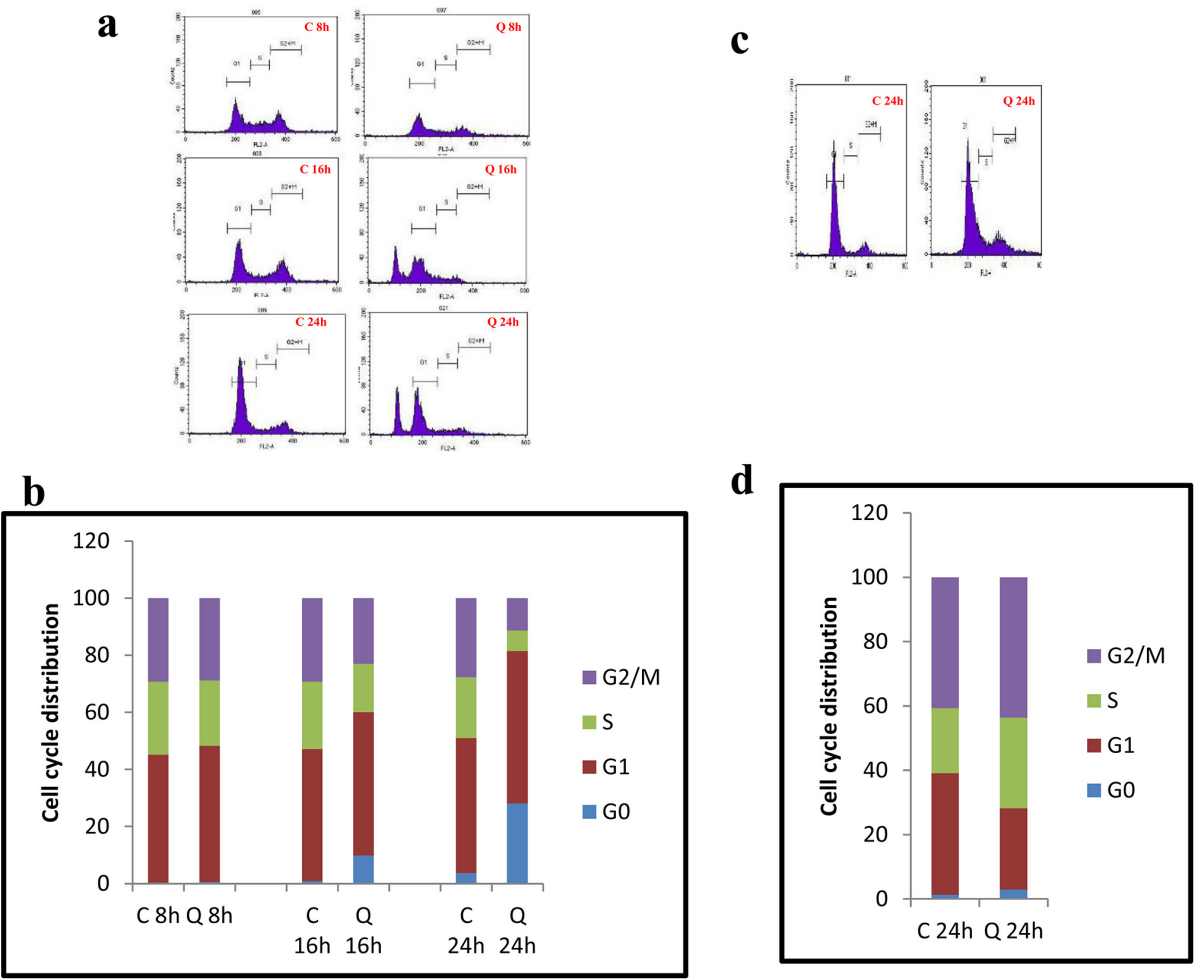
Flow cytometry analysis showing the effect of quercetin on cell cycle. A. MCF-7 cells were treated with 40μM quercetin for 8h, 16h and 24h and then harvested for cell cycle analysis. The picture represents the distribution of MCF-7 cells in different phases of cell cycle. B. Bar graph represents the percentage distribution of MCF-7 cells in different phases of cell cycle. C. MDA-MB-231 cells were treated with 40μM quercetin for 24h and then harvested for cell cycle analysis. The picture represents the distribution of the cells in different phases of cell cycle. D. Bar graph represents the percentage distribution of MCF-7 cells in different phases of cell cycle. The cells were plated at a density of 1x10^5^ cells/dish and exposed to quercetin for various time periods. Cells were harvested and analysed for cell cycle. The cells with DMSO exposure served as control. The significant values represent mean ± SD of three separate experiments, each consisting of duplicate cultures.

MCF-7 cells exposed to 40μM of quercetin for 8h, 16h and 24h showed a gradual increase in G1 phase which implies the apoptotic dead cells. The effect of quercetin was more prominent in 24h of exposure. Quercetin treatment increased the cells at G1 phase, 8h, 16h and 24h of exposure showed 47.66%, 50.12% and 53.35%, respectively compared to the control which was 44.79%, 46.29% and 47.21%, respectively. This indicates a time dependant increase in apoptosis of MCF-7 cells by quercetin (Fig 4A), which was shown graphically in Fig 4B.

In case of MDA-MB-231 cells exposed to 24h of quercetin alone showed equal distribution of cells in all phases were seen. However, there was more number of cells in S phase when compared to the control cells. The cells at S phase were 28.12% for 20.17% control, in 24h of quercetin treatment. Significantly increased cell population was seen in S phase at selected dose of quercetin indicating continuous proliferation of MDA-MB-231 cells (Fig 4C), which was shown graphically in Fig 4D.

### Expression analysis of proteins involved in cell cycle arrest

**Fig 5:**
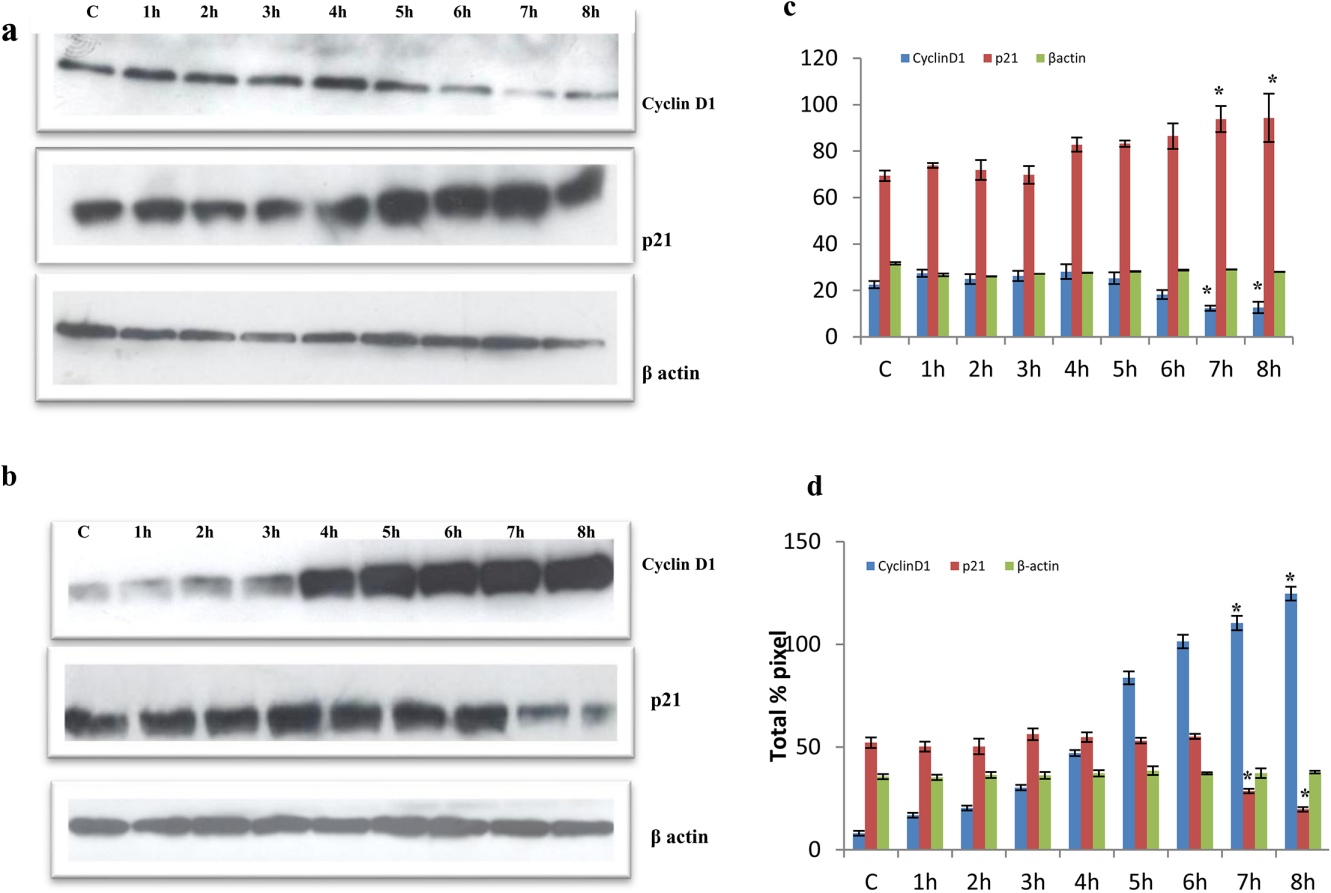
Quercetin regulates cell cycle regulatory proteins. A. Protein expressions of Cyclin D1 and p21 were assessed by western blotting in MCF-7 cells treated with quercetin at different time intervals. β-actin was used as an internal control. c. Quantitative data of a. B. Protein expressions of Cyclin D1 and p21 were assessed by western blotting in MDA-MD-231 cells treated with quercetin at different time intervals. β-actin was used as an internal control. d. Quantitative data of b. The significant values represent mean ± SD of three separate experiments, each consisting of duplicate cultures.

As there is a G1 arrest in quercetin treated cells, we examined the gene expression levels of cyclinD1 and p21 in MCF-7 cells. Our findings demonstrated that quercetin treated MCF-7 cells showed a gradual decrease in cyclin D1 where the control cells expressed more of cyclinD1. Similarly, the level of p21 expression was increased on treatment with quercetin, indicating the effect of quercetin on cell cycle regulatory molecules (Fig 5A).

Inversely, in case of MDA-MB-231 cells, cyclinD1 expression was significantly elevated on quercetin treatment when compared to the control. Along with this, the expression of p21 decreased on exposure of quercetin, which indicates that cells continue to proliferate even on quercetin treatment (Fig 5B).

### Effect of quercetin in expression of Twist and phospho p38MAPK

**Fig 6:**
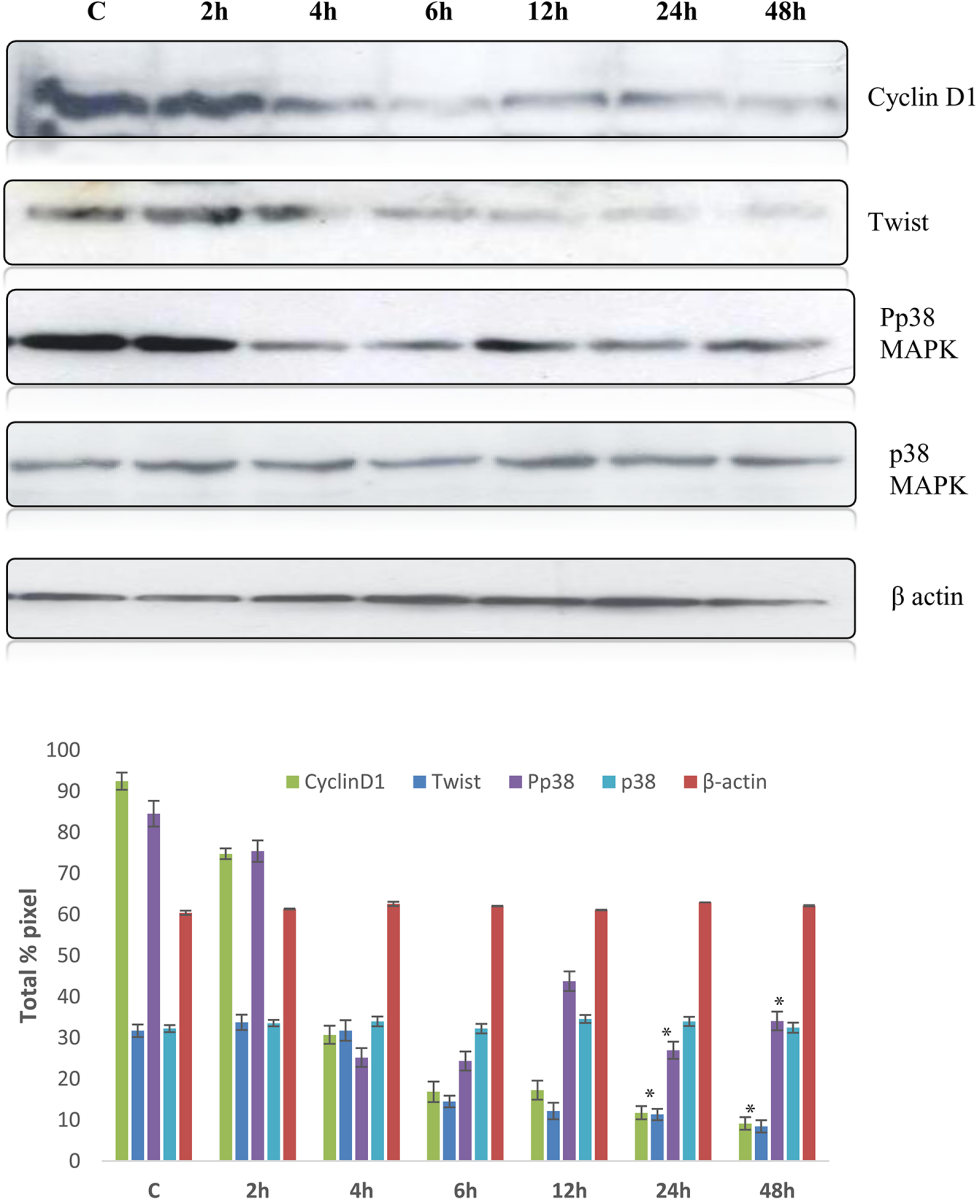
Effect of quercetin in CylinD1, twist and p38MAPK in MCF-7 cells. A. Expression of Cyclin D1, twist and Pp38 MAPK were assessed by western blot MCF-7 cells with quercetin. The significant values represent mean ± SD of three separate experiments, each consisting of duplicate cultures. B. Quantitative analysis of a.

**Fig 7:**
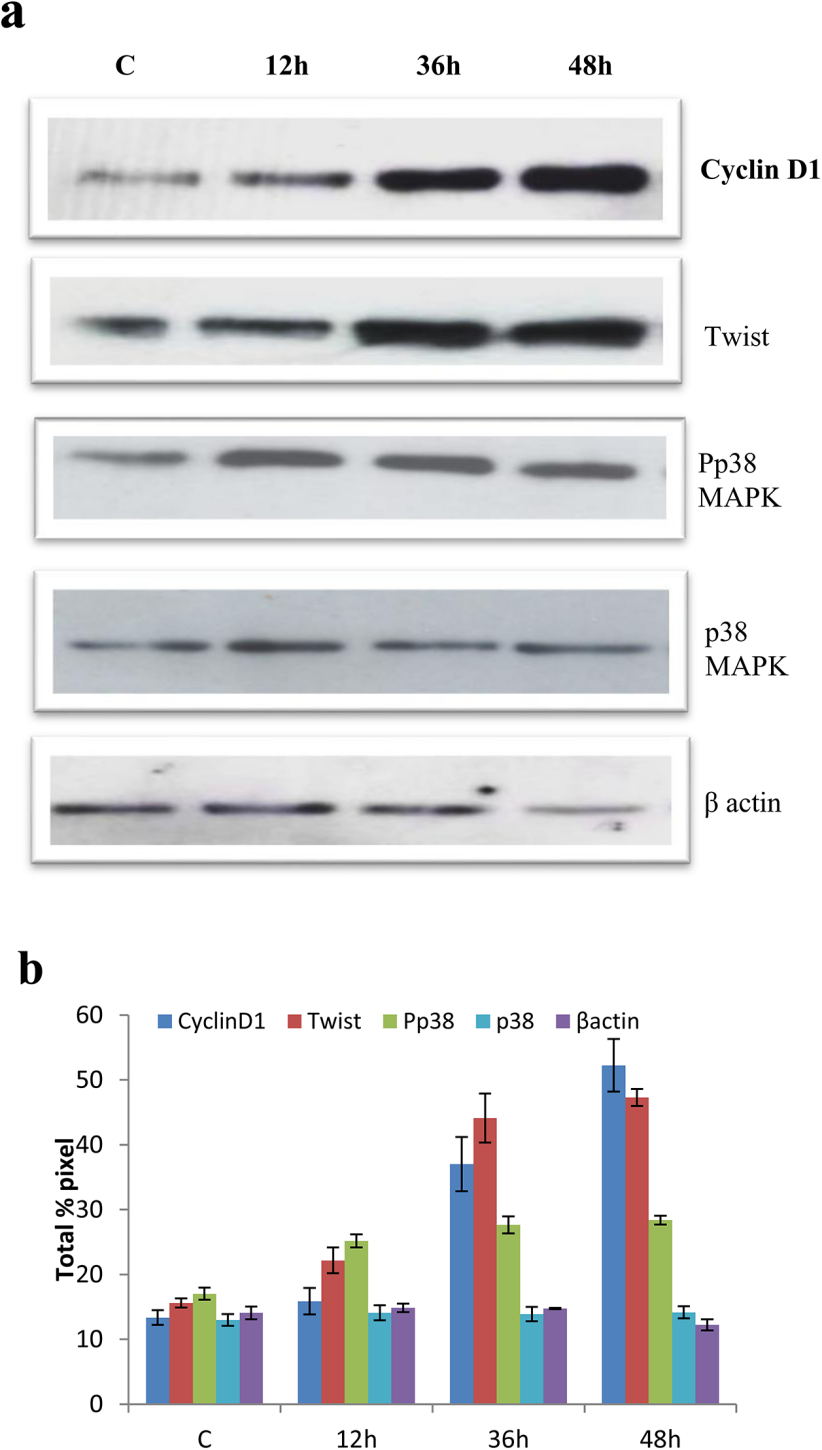
Effect of quercetin in cylinD1, twist and p38MAPK in MDA-MB-231 cells. A. Expression of Cyclin D1, twist and Pp38 MAPK were assessed by western blot in MDA-MB-231 cells with quercetin. B. Quantitative analysis of a. The significant values represent mean ± SD of three separate experiments, each consisting of duplicate cultures.

In the present study, quercetin effectively inhibited the growth of breast cancer cells, hence we analysed the ability of quercetin in suppression of twist in MCF-7 cells. Our results showed that quercetin efficiently suppressed the expression of twist, when compared to control (Fig 6A) which was also confirmed by immunofluorescence (Fig 6B). Similarly, phosphorylation of p38MAPK was decreased in quercetin exposed cells when compared to the phosphorylated p38MAPK in control MCF-7 cells, indicating the possible mechanism of quercetin in inhibiting the cancer growth through p38MAPK pathway (Fig 6A). However, in MDA-MB-231 cells, twist and phospho p38MAPK were increased when compared to control even after quercetin treatment (Fig 7A). The expression of twist was further confirmed by immunofluorescence assay (Fig 7B).

### Over-expression of twist in MCF-7 cells

**Fig 8:**
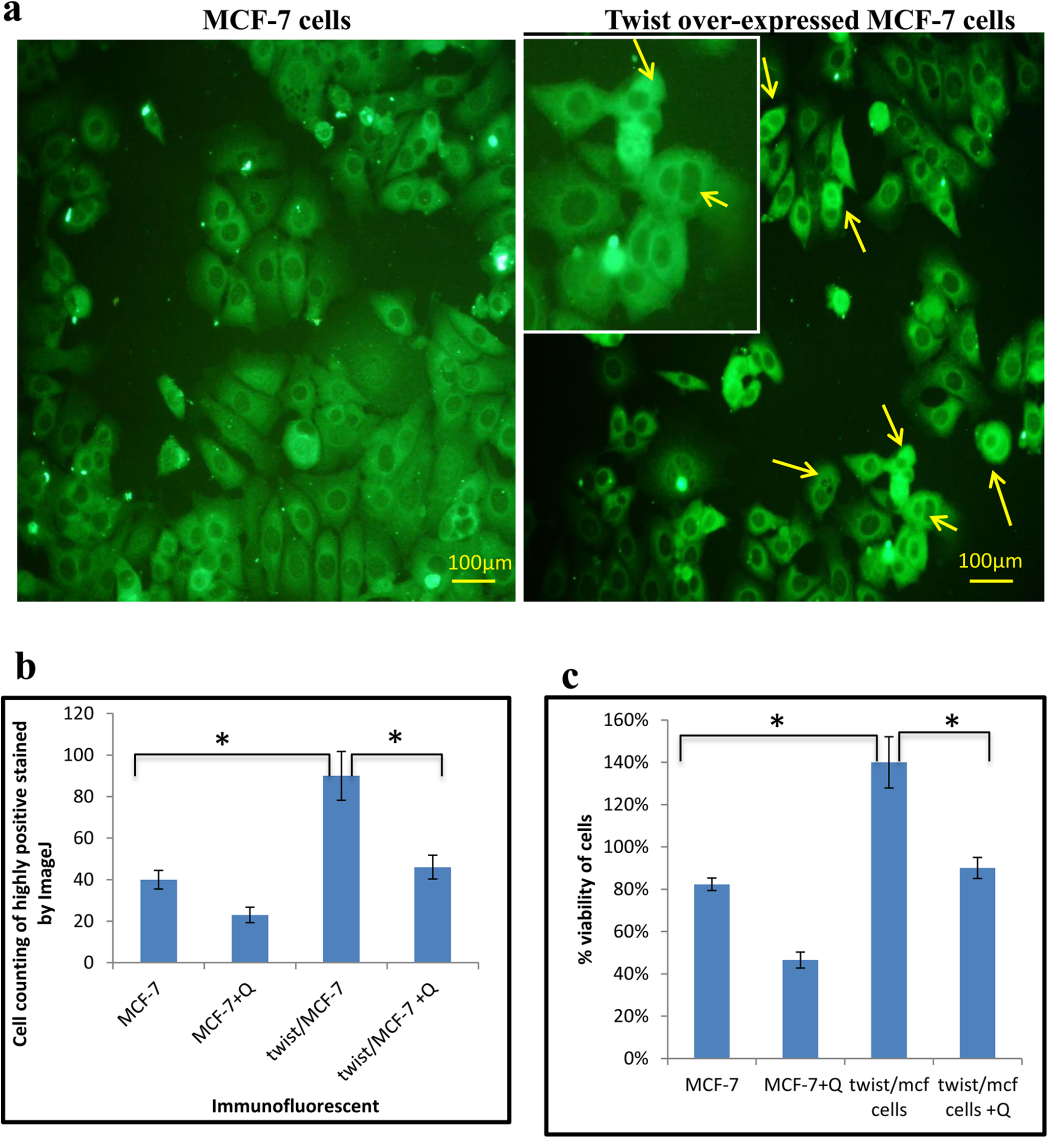
Twist over-expression induced cell proliferation. A. Twist plasmid was transfected in MCF-7 cells to over-express twist. Levels of twist expression were assessed by immunofluorescence, in control and twist over-expressed MCF-7 cells. The twist over-expressed MCF-7 cells showed more number of dividing cells which was not observed in control MCF-7 cells. B. Number of highly positive stained cells in five random fields was counted by ImageJ. C. Viability of cells (%) assessed by MTT assay in control and twist over-expressed MCF-7 cells. The significant values represent mean ± SD of three separate experiments. Significance is indicated as *p<0.05.

To determine the molecular mechanism of quercetin in regulating twist, it was over-expressed using a plasmid construct (pEF1 Myc-His) which expressed full length twist (gift from Dr. Venu Raman, JHMU, USA). When we analysed the expression levels of twist protein in transfected MCF-7 (twist/MCF-7) cells by immunofluorescence, interestingly there were more number of dividing cells which was not seen in control (Fig 8A). This indicates the involvement of twist in inducing cell proliferation. The highly positive stained cells were measured using ImageJ software, which showed a significant intense stained cells in twist/MCF-7 cells when compared to control. Quercetin treatment significantly reduced the number of stained cells (Fig 8B), indicating the effect of quercetin in suppression of twist. Similarly, the viability of twist/MCF-7 cells was increased, which was reduced by quercetin treatment (Fig 8C).

**Fig 9:**
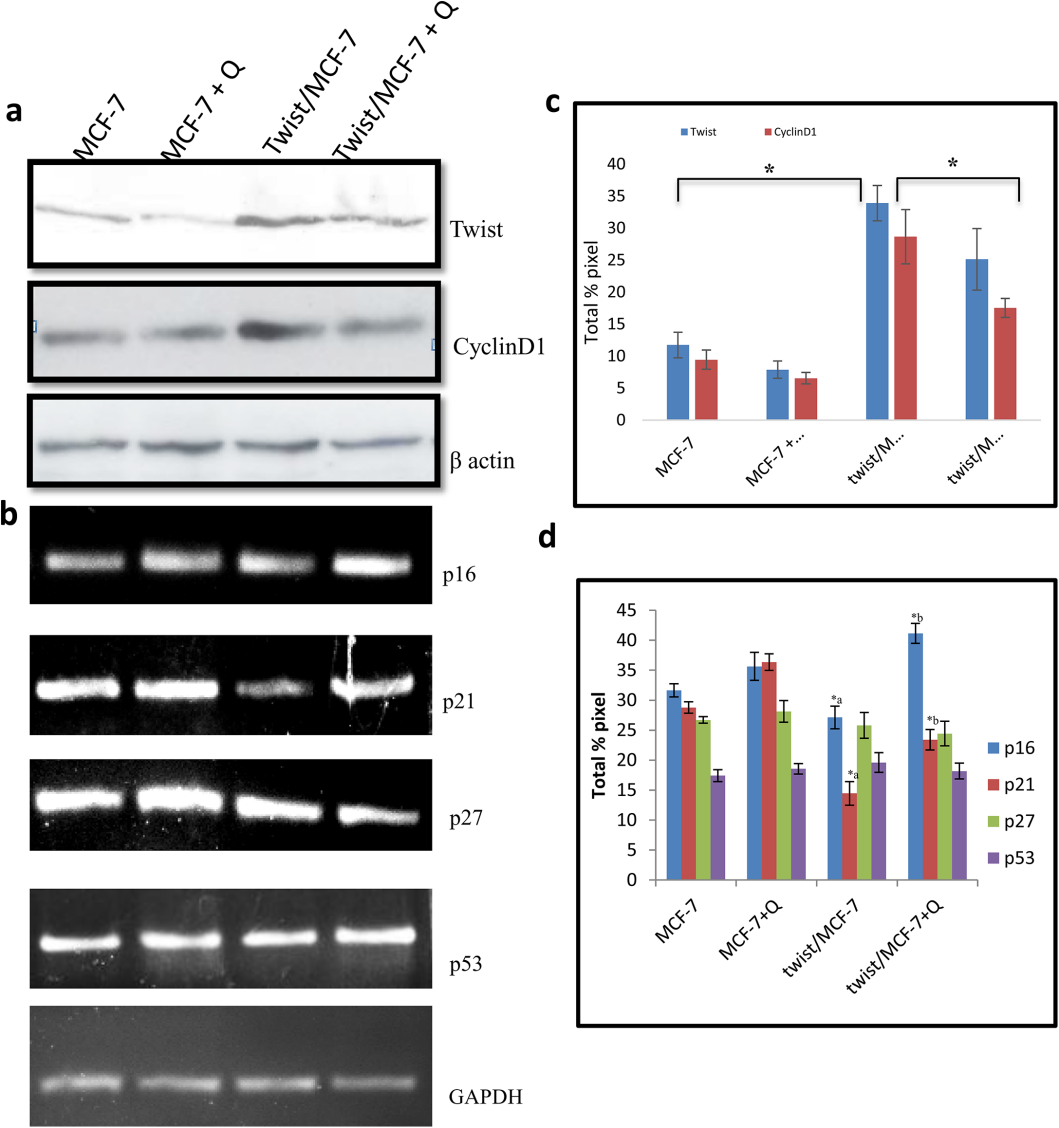
Inhibitory effect of quercetin post-transfection. A. Twist and Cyclin D1 expression levels were assessed by western blot in twist over-expressed MCF-7 cells and the same after Q treatment. c. quantitative analysis of a. B. Expression of p16, p21, p27 and p53 were analyzed by RT-PCR in twist over-expressed MCF-7 cells and the same after Q treatment. d. Quantitative analysis of b. The significant values represent mean ± SD of three separate experiments. Significance is indicated as *p<0.05.

Quercetin significantly suppressed the levels of twist in twist/MCF-7 cells along with the suppression of cyclinD1 expression, which indicates that quercetin is efficient in regulating twist (Fig 9A). Together, when we analysed the expression of cell cycle regulatory molecules p16, p21, p27 and p53, quercetin induced the expression of p16 and p21 for the cells to get arrested in G1 phase (Fig 9B). However, there were no significant changes observed in expression of p27 and p53, suggesting the role of p16 and p21 in quercetin induced cell cycle arrest.

### Ability of quercetin in reducing the malignancy of cancer cells

**Fig 10:**
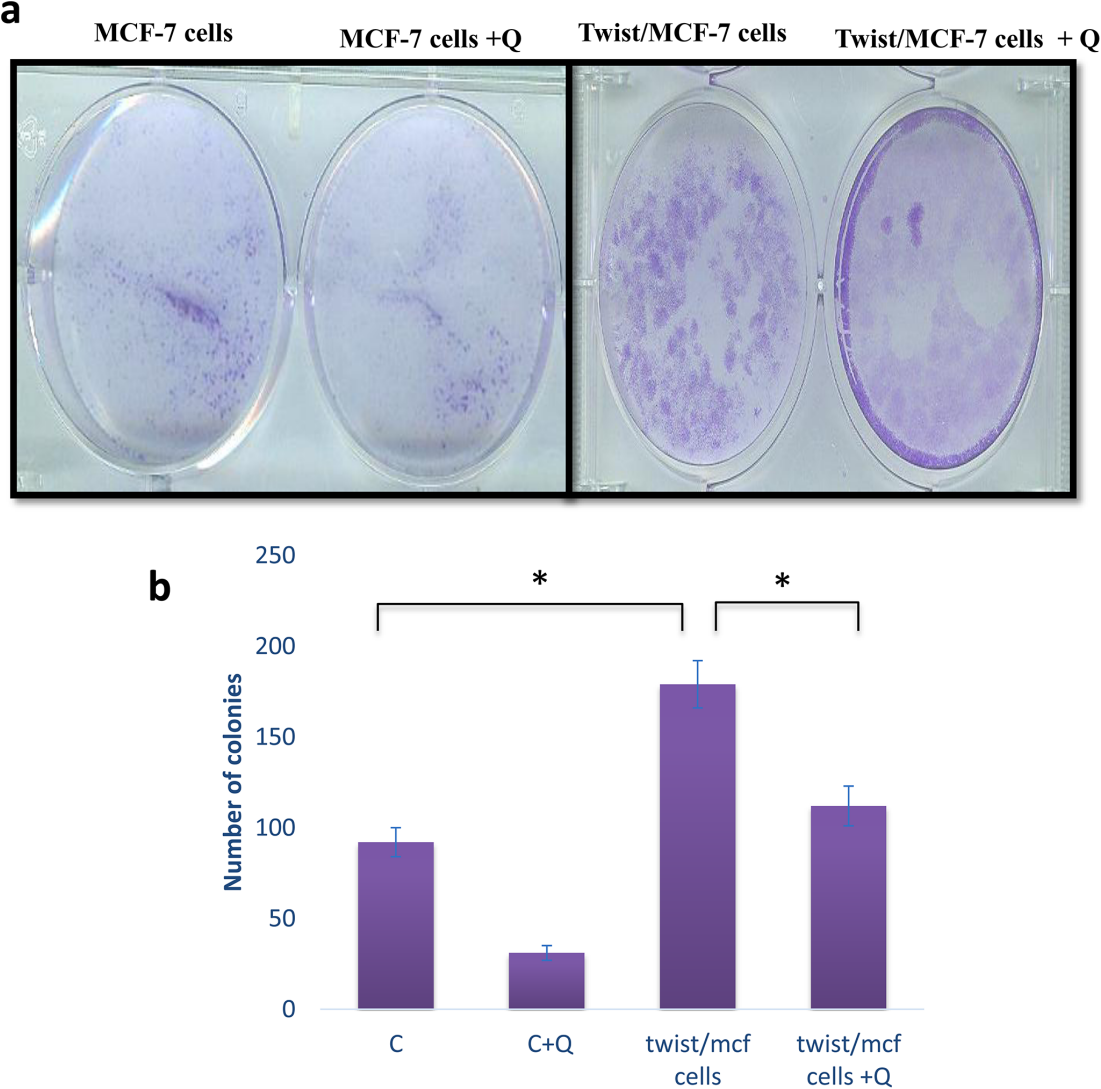
Quercetin inhibits anchorage-independent growth. A. In soft agar assay, colonies formed in top agar layer by cancer cells after 20 days incubation were stained with 0.005% crystal violet and counted under a light microscope. B. The number of colonies formed after treatment with quercetin compared with those formed by twist/MCF-7 cells is shown at the graph and the twist/MCF-7 compared with control MCF-7 which are statistically significant ‘*‘ as determined by Student’s t-test. The significant values represent mean ± SD of three separate experiments, each consisting of duplicate cultures. Significance is determined as *p<0.05.

To further investigate the hypothesis that quercetin could target twist protein and reduce the oncogenic potential of cells, we performed soft agar colony assay. Viability assay showed that the induction of twist, increased cell growth and doubled the cell number in twist/MCF-7 cells when compared to control MCF-7 cells (Fig 8C). However, treatment with quercetin significantly reduced the cell viability and notably the cell numbers approached the levels of control cells. Furthermore, the over-expression of twist increased the ability of MCF-7 cells to form larger colonies in soft agar, but quercetin treated cells reduced anchorage-independent growth (Fig 10A), the number of colonies were counted and plotted graph (Fig 10B). These findings suggest that quercetin reduces the oncogenic property of breast cancer cells.

## Discussion

Breast cancer is the most common cancer among women in India and worldwide. There are several chemotherapeutic agents available for breast cancer treatment, which weaken and destroy fast dividing cancer cells. It could also harm the normal fast dividing cells like hair follicles, nail, digestive tract and bone marrow, possibly causing side effects. On the other hand, hormone therapy, used as an adjuvant therapy reduce the risk of breast cancer [29], however the use of this therapy lower estrogen levels or stop estrogen acting on cancer cells which leads to side effects like bone thinning and osteoporosis. Moreover, chemotherapy resistance has been considered a major cause of tumor recurrence, leading to the failure of clinical treatment.

A natural drug could overcome all the side effects caused, in most cases effective on the cancer cells without harming the normal cells. However, the molecular mechanism behind the action of the drug is not well defined. Quercetin is one such flavonoid which acts as phytoestrogen, used as an anti-inflammatory, anti-oxidant and also anti-cancer agent whose mechanism of action is still under debate [16]. Although studies say that quercetin may protect against some forms of cancer when consumed in the diet [30, 31]. However, there are also few studies which state that higher dosage of quercetin is carcinogenic and increases the risk of leukemia in young children [27, 28]. Hence in this study, we restricted to lower dosage of quercetin. Most of the breast cancers are estrogen receptor positive and estrogen plays an important role in cancer cell growth, survival, as well as in gene expression regulatory mechanism by binding to estrogen receptor (ER) [32]. Thus, the role of the presence of estrogen receptor in the molecular mechanism of quercetin on breast cancer cells was studied using two breast cancer cell lines MCF-7 and MBA-MD-231 which are ER positive and ER negative, respectively.

Quercetin exerted cytotoxicity in dose dependant manner in MCF-7 cell line when compare to MDA-MB-231cells. IC_50_ value of MCF-7 cells was lower 37μM, however there was no significant cytotoxicity observed in MDA-MB-231cells even at 100μM of quercetin treatment. Hence, further study was performed by treating cells with a fixed concentration of 40μM for both the cell lines to assess the difference in cellular events on quercetin treatment. Further, dual staining assay with acridine orange and ethidium bromide confirmed that cytotoxicity of the selected dosage of quercetin in MCF-7 cell line was due to apoptosis. However, MDA-MB-231 cells showed more number of viable cells at the selected concentration. Damaged cells with high chromatin condensation, autophagosis and more vesicles in the cytosol by Transmission electron microscopy (TEM) which confirm that quercetin induced apoptosis of MCF-7 cells.

In many cancer cells, quercetin is known to induce apoptosis through cell cycle arrest by modulating various cell cycle regulators including p21, p27 [33]. The cell cycle analysis suggests that more number of apoptotic cells were observed in 16h and 24h of quercetin treatment when compared to the control MCF-7 cells. Along with the apoptotic cells, G1 check point arrest was also noticed in 24h of quercetin treatment in MCF-7 cells. On the other hand, there was no significant apoptotic cells observed in MDA-MB-231 cells even at 24h of quercetin treatment. Interestingly, more number of cells were found in S phase when compared to the control MDA-MB-231 cells.

The expression of cell cycle regulator molecule cyclinD1 was analysed as there was an arrest in G1 check point in cell cycle. Quercetin treated MDA-MB-231 cells showed an increase in cyclin D1 expression compared to its control. Cyclin D1 expression was down regulated in quercetin treated MCF-7 cells when compared to the control, suggesting the role of quercetin in regulation of cell cycle regulatory molecule Cyclin D1. Accumulation of Cyclin D1 leads to cell cycle disregulation favouring cell proliferation and survival as seen in many cancers [34]. Quercetin causes cell cycle arrest by decreasing levels of Cyclin D1 causing accumulation of cells at G1 phase which leads to G1/S check point arrest.

Cyclin D1 expression was regulated by diverse signal transduction events including Wnt pathway, Akt pathway [35, 36]. More recently, few studies revealed the direct and indirect role of Twist in inhibition of a tumor suppressor E-Cadherin [3]. It is well known that inhibition of E-Cadherin will cause accumulation of β-catenin within cells leading to nuclear translocation. Entry of β-Catenin into nucleus activates expression of Cyclin D1 by binding to its promote [37, 38]. Twist is a basic helix-loop-helix (bHLH) transcription factor, which is a key transcription activator of epithelial to mesenchymal transition (EMT) and it is observed in various cancer cell lines including breast cancer [3, 39, 40]. Increased twist levels in quercetin treated MDA-MB-231 cells promoted cell proliferation by increasing Cyclin D1. Decrease in levels of Twist by quercetin induced apoptosis in MCF-7 cells through cell cycle arrest by down regulating Cyclin D1.

Phospho p38MAPK plays a vital role in cell proliferation in the many cancers including breast [41]. Recent studies showed that phospho p38MAPK stabilizes Twist by phosphorylating twist at serine 68 [42]. Decrease in twist in quercetin treated MCF-7 cells is due to decreased phospho p38MAPK suggesting quercetin acts through p38MAPK. However, efficacy of quercetin to down regulate the expression of Twist cannot be neglected.

To determine the molecular mechanism of quercetin in regulating twist, it was over-expressed in MCF-7 cells using a plasmid construct (pEF1Myc-His), gifted by Dr.Venu Raman, Johns Hopkins Medical University, USA. Over-expression of twist induced cell proliferation through Cyclin D1 up-regulation which in turn increased the tumorigenic property of MCF-7 cells, indicating the involvement of twist in breast cancer progression. Quercetin effectively reduced the levels of twist thereby inhibiting cell proliferation through down-regulation of Cyclin D1 which reduced the tumorigenic property of twist/MCF-7 cells.

**Fig 11:**
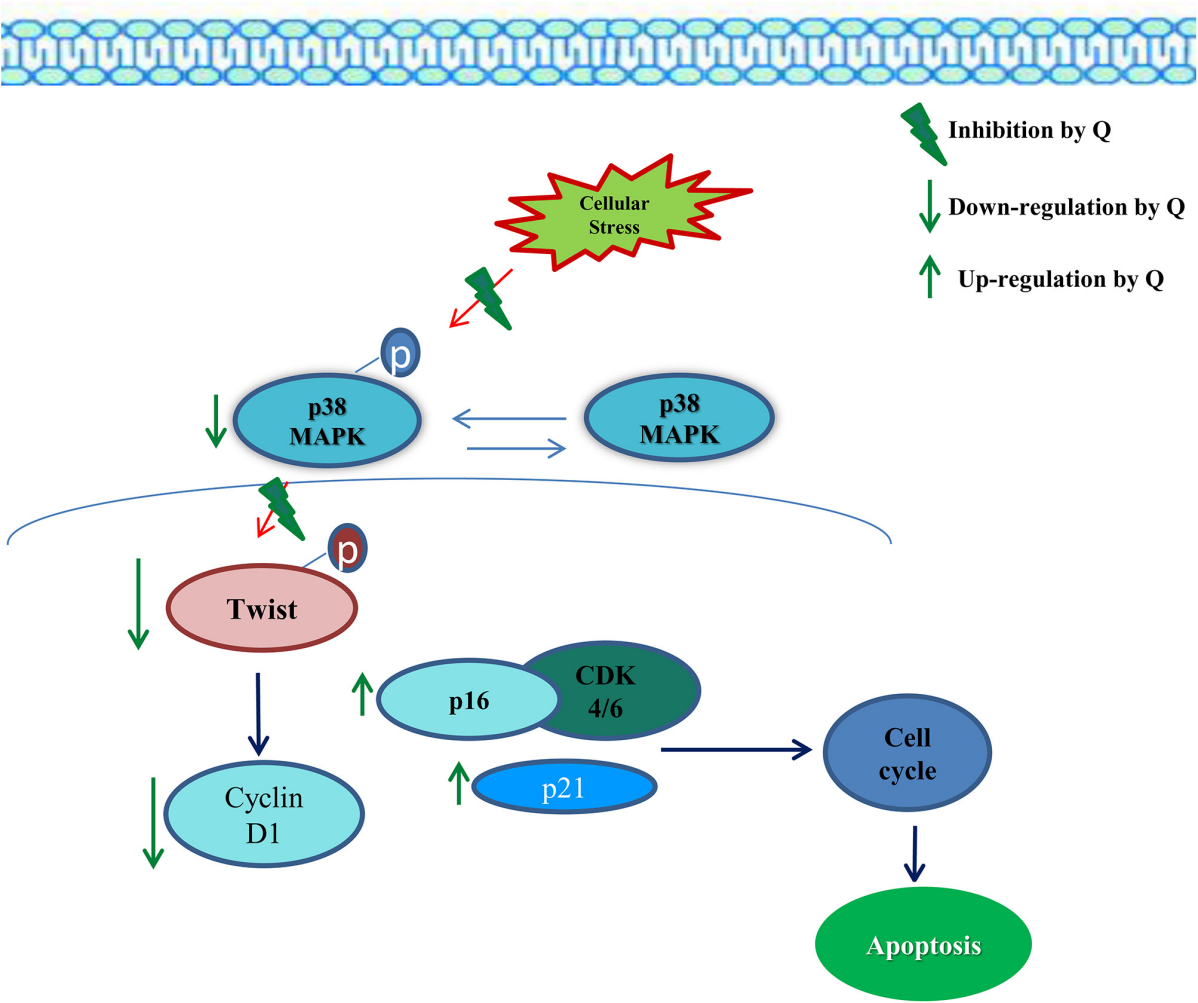
Schematic representation of quercetin induced apoptosis through twist regulation. Quercetin being a potent anti-oxidant it reduces the cellular stress. This indirectly reduced the activation of p38MAPK in survival pathway which prevents the stabilization of twist. Quercetin reduced twist gene expression through suppression of Cyclin D1, and induced p16 and p21 to arrest the cells at G1 phase of cell cycle, thereby induced apoptosis. Our data thereby reveal the mechanism of quercetin induced apoptosis through twist regulation.

Cell division depends on the binding of cyclin D1 to cyclin-dependent kinases (CDKs) to induce cell cycle progression towards S phase and later to initiate mitosis. But its function is tightly regulated by few cell-cycle inhibitors including p16, p21 and p27 proteins, where they bind to cyclin–CDK complexes to inhibit their catalytic activity and induce cell-cycle arrest. Quercetin induced over expression of p16 and p21 is due to down regulation of twist, which was reverted in quercetin treated twist over expressed MCF-7 cells. It is well known that twist repress p21 and p16 transcription [43]. To conclude, Quercetin, down regulates twist and induces apoptosis through cell cycle arrest in MCF-7 cells (Fig 11).

There was no significant inhibition of cell growth observed in MDA-MB-231 cells at the selected dosage of quercetin, though few studies showed cytotoxicity of quercetin at higher dose [44]. However, as Li N *et al* states, lower dose of quercetin might be effectively used in combination with other therapeutic agents which reduces the therapeutic side effects caused [45, 46]. Thus, quercetin acts as a potential anti-breast cancer agent in estrogen receptor positive breast cancer.

## Supporting Information

S1 Fig Effect of quercetin in cell viability in human breast cancer MCF-7 cells. The percentage of cell viability was determined by trypan blue exclusion test. MCF-7 cells were treated with 40μM quercetin for 8h, 16h and 24h and then harvested. To 1ml of the cells, 0.1 ml of 0.4% trypan blue was added. The number of blue stained cells and total number of cells were counted using haemocytometer. % viable cells = [1.00 –(Number of blue cells ÷ Number of total cells)] x 100.
(TIF)

S2 Fig Effect of quercetin in cell viability in human breast cancer MDA-MB-231 cells. The percentage of cell viability was determined by trypan blue exclusion test. MDA-MB-231 cells were treated with 40μM quercetin for 24h and then harvested. To 1ml of the cells, 0.1 ml of 0.4% trypan blue was added. The number of blue stained cells and total number of cells were counted using haemocytometer. % viable cells = [1.00 –(Number of blue cells ÷ Number of total cells)] x 100.
(TIF)

S1 Table List of primer sequences used for RT-PCR. (TIF)
