# Supplementary figures and images for: Quercetin Suppresses Twist to Induce Apoptosis in MCF-7 Breast Cancer Cells
Source: PLoS One. 2015 Oct 22;10(10):e0141370. doi: 10.1371/journal.pone.0141370 (PMC4619597; doi:10.1371/journal.pone.0141370)

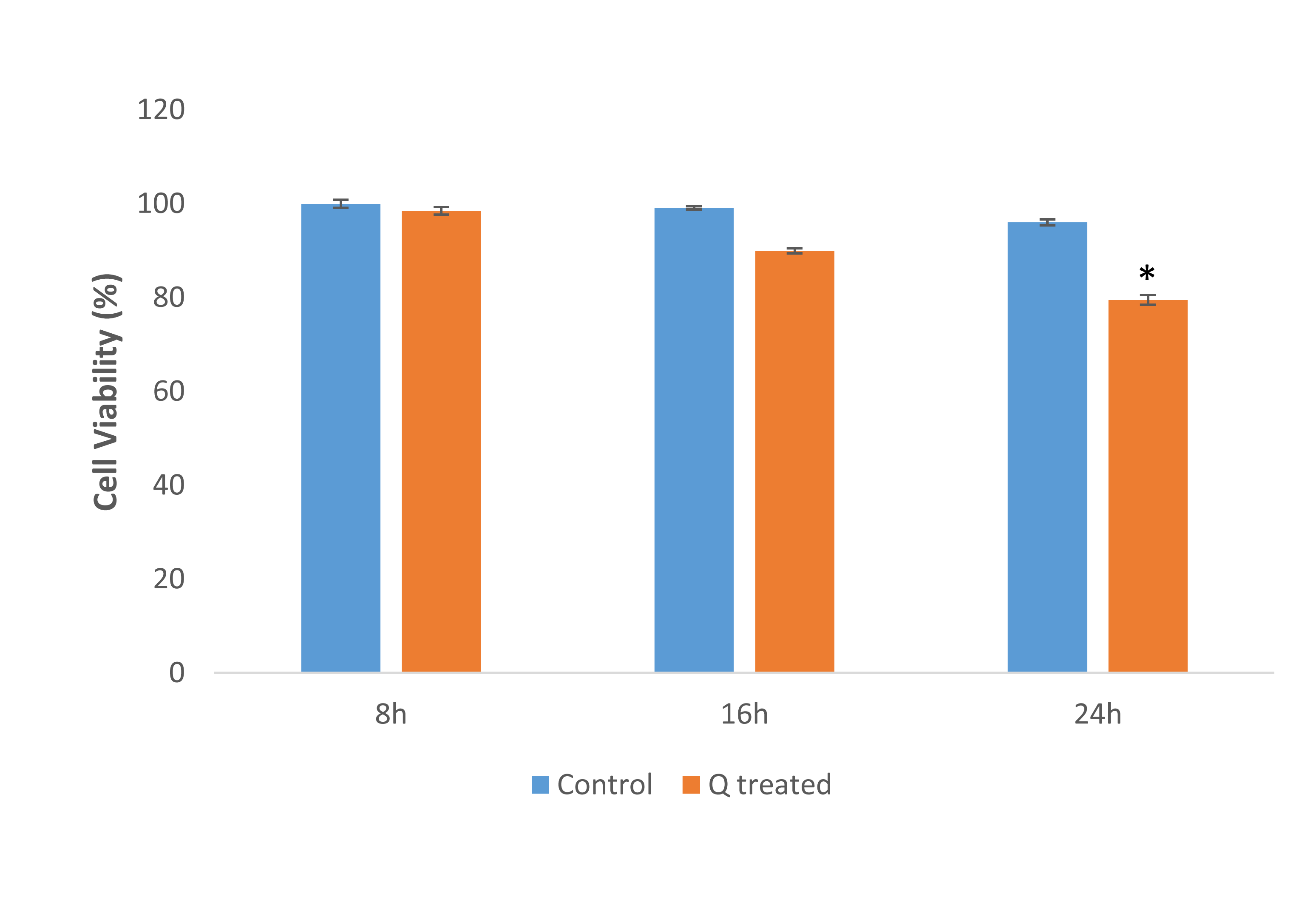

Supplement: S1 Fig — The percentage of cell viability was determined by trypan blue exclusion test. MCF-7 cells were treated with 40μM quercetin for 8h, 16h and 24h and then harvested. To 1ml of the cells, 0.1 ml of 0.4% trypan blue was added. The number of blue stained cells and total number of cells were counted using haemocytometer. % viable cells = [1.00 –(Number of blue cells ÷ Number of total cells)] x 100. (TIF) [file pone.0141370.s001.tif]

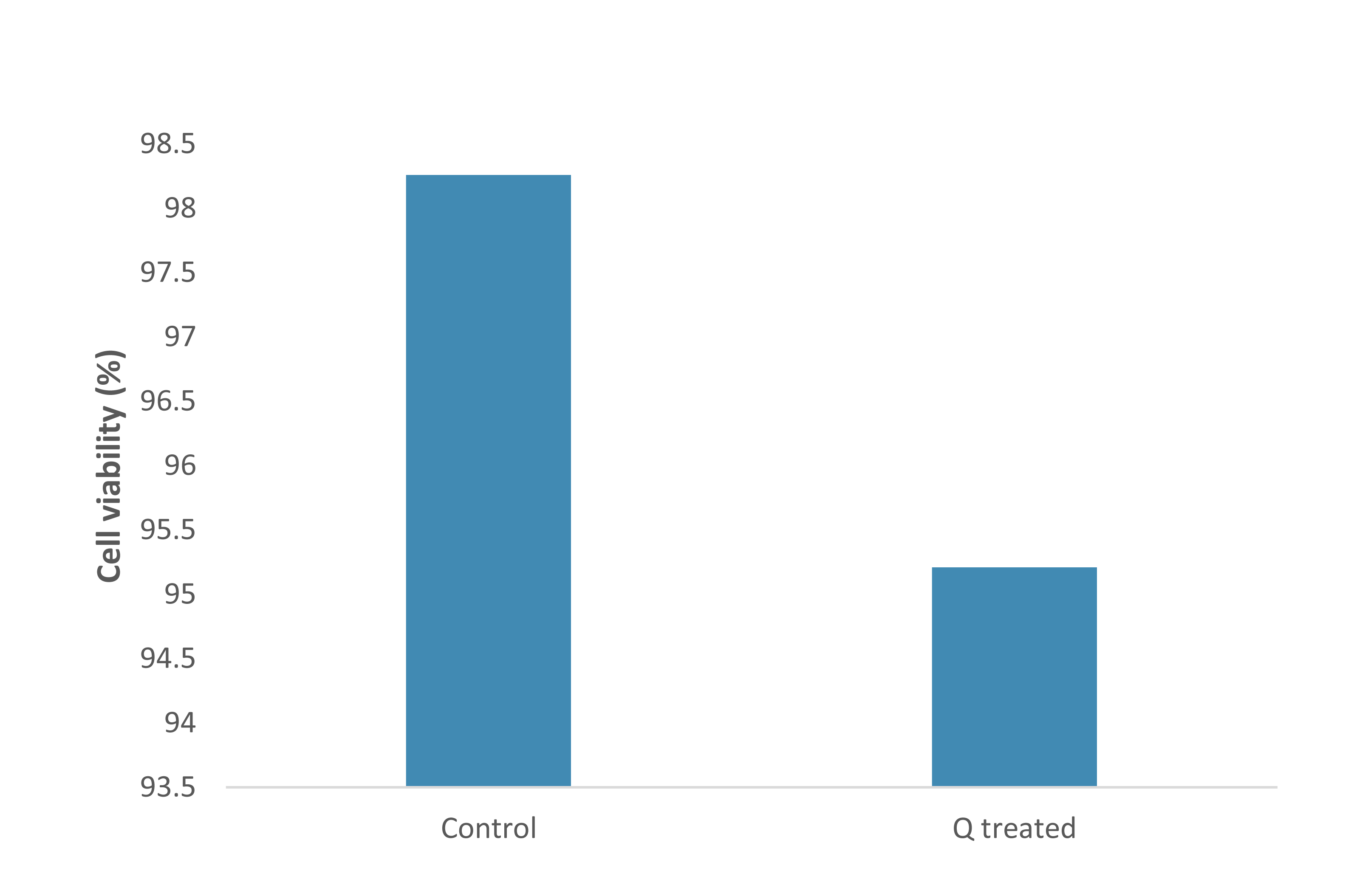

Supplement: S2 Fig — The percentage of cell viability was determined by trypan blue exclusion test. MDA-MB-231 cells were treated with 40μM quercetin for 24h and then harvested. To 1ml of the cells, 0.1 ml of 0.4% trypan blue was added. The number of blue stained cells and total number of cells were counted using haemocytometer. % viable cells = [1.00 –(Number of blue cells ÷ Number of total cells)] x 100. (TIF) [file pone.0141370.s002.tif]

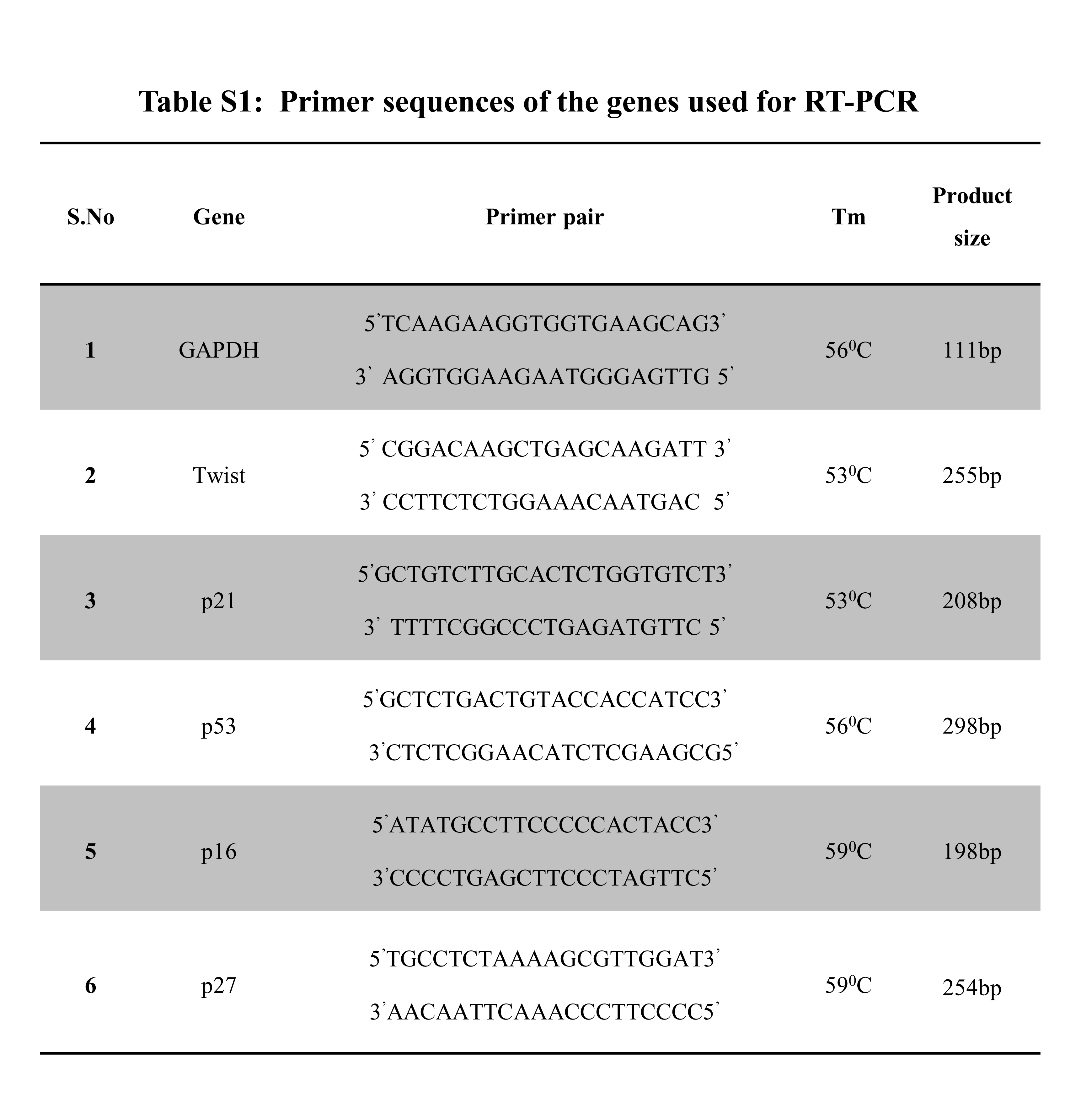

Supplement: S1 Table — (TIF) [file pone.0141370.s003.tif]
